# Association of Autophagy-Related Gene 5 (ATG5) With Neonatal Cholestasis in Egyptian Pediatric Patients

**DOI:** 10.30699/ijp.2025.2061573.3466

**Published:** 2025-08-15

**Authors:** Rehab M Samaka, Hala S El-Rebey, Asmaa M Kabouh, Shereen M El- Mashad

**Affiliations:** 1 *Pathology Department, Faculty of Medicine, Menoufia University, Shebin Elkom, Menoufia, Egypt*; 2 *Pathology Department, National Liver Institute, Menoufia University, Shebin Elkom, Menoufia, Egypt*

**Keywords:** ATG5, neonatal cholestasis, immunohistochemistry, biliary atresia, jaundice

## Abstract

**Background & Objective:**

Neonatal cholestasis (NC) occurs in approximately 1 in 2500 live births. Autophagy-related gene 5 (ATG5) is a central component of the autophagy machinery, particularly in autophagosome formation. The autophagic process regulated by ATG5 has been implicated in various physiological and pathological conditions. This study aimed to evaluate the role of ATG5 in NC.

**Methods:**

This retrospective study analyzed liver biopsies from 74 patients with NC. 46 with extrahepatic etiology and 28 with intrahepatic etiology. Immunohistochemical expression of ATG5 was assessed in hepatocytes and biliary epithelium.

**Results:**

A significant association was observed between intrahepatic cholestasis and the intensity of ATG5 expression in zone II hepatocytes (P = .029). Overexpression of ATG5 in hepatocytes was significantly associated with mild portal tract fibrosis (P = .038) and mild lymphocytic infiltrates (P = .005).

**Conclusion:**

ATG5 appears to contribute to the pathogenesis of NC in Egyptian infants. These findings may provide a basis for further research into novel diagnostic and therapeutic strategies.

## Introduction

The incidence of neonatal cholestasis (NC) is approximately 1 in 2,500 live births worldwide. At the Egyptian National Liver Institute, the most common cause of NC was biliary atresia (BA) (37%), followed by progressive familial intrahepatic cholestasis (PFIC) (12%), neonatal sepsis (9%), cytomegalovirus (CMV) infection (8%), idiopathic neonatal hepatitis (7%), and inspissated bile syndrome (3%) (2).

Autophagy is a catabolic process that allows cells to recycle amino acids and other intracellular components to generate energy (3). In the liver, autophagy has been shown to play a crucial role in cytoprotection against various pathological insults, including steatosis, liver injury, and dyslipidemia associated with alcoholic and nonalcoholic fatty liver disease (4). Furthermore, autophagy deficiency has been reported in inherited metabolic liver diseases, such as glycogen storage disease type Ia (GSD-Ia), which is characterized by a deficiency in glucose-6-phosphatase-α (G6Pase-α), leading to impaired glucose homeostasis and hepatomegaly.

Autophagy-related genes were first identified in yeast; among them, autophagy-related gene 5 (ATG5) is a key regulator of autophagosome formation (5).

The present study aimed to evaluate the role of ATG5 in NC among Egyptian pediatric patients.

## Materials and Methods

This retrospective study included 74 cases of neonatal cholestasis (NC): 46 with extrahepatic etiology and 28 with intrahepatic etiology. Cases were collected over a 4-year period (January 2017–December 2020) from the Pathology Department of the National Liver Institute, Menoufia University. 

Patients were excluded if paraffin blocks were unavailable or contained insufficient tissue, if clinical data were incomplete, or if treatment and follow-up were conducted outside the National Liver Institute.

### Clinical and Laboratory Data

Laboratory and radiological findings were obtained from patient records. Laboratory data included prothrombin time, international normalized ratio (INR), liver function tests [aspartate aminotransferase (AST), alanine aminotransferase (ALT), γ-glutamyl transferase (GGT), alkaline phosphatase (ALKP), total and direct bilirubin, total protein, and albumin]. Abdominal ultrasound results were also reviewed.

### Histopathological Evaluation

Hematoxylin-eosin (H&E)–stained sections and special stains (Masson trichrome, Perls, and orcein) were re-evaluated to confirm the diagnosis. The following pathological parameters were graded: ductular proliferation, bile plugs, portal inflammatory infiltrates (lymphocytes, neutrophils, and eosinophils), giant cell transformation of hepatocytes, hepatocyte swelling, cholestatic rosettes, and extramedullary hematopoiesis (6,7). Liver fibrosis was staged according to the Ishak system (grades 0–6) (8).

### Immunohistochemistry

Four-micron sections were prepared from paraffin blocks and stained using a streptavidin-biotin amplification system. Sections were deparaffinized in xylene, rehydrated, and treated with 200 mL of Tris-EDTA high-pH retrieval solution (Dako, Ref K8000, Glostrup, Denmark) for 20 minutes. Endogenous peroxidase activity was blocked using peroxidase-blocking reagent. Slides were incubated overnight with primary antibody against ATG5 (goat polyclonal, Santa Cruz Biotechnology, catalogue no. sc-8667, RRID: AB_2062328; dilution 1:200). Normal human duodenal tissue served as a positive control. Sections incubated without primary antibody served as negative controls.

Immunohistochemical results were evaluated independently by two pathologists blinded to clinical data, with consensus obtained in cases of discrepancy.

- **Hepatocytes:** ATG5 positivity was defined as diffuse granular cytoplasmic staining (9). The H-score was calculated using the formula:H-score = (1 × % mildly stained cells) + (2 × % moderately stained cells) + (3 × % strongly stained cells).Staining was assessed separately across hepatocyte zones.
- **Biliary epithelium:** Immunoreactivity was scored using the IRS system (9). Staining intensity was scored as 0 (negative), 1 (faint), 2 (moderate), or 3 (strong). Distribution was scored as 0 (negative), 1 (1%–30%), or 2 (31%–100%). The composite score was calculated by multiplying intensity and distribution scores. Scores of 0 were considered negative, while scores 1–6 indicated positive expression.

### Statistical Analysis

Data were analyzed using SPSS version 20 (IBM Corp, Armonk, NY). Qualitative variables were described as frequency and percentage, and quantitative variables as mean ± standard deviation or median (range) as appropriate. The χ² test or Fisher exact test was used for categorical variables. Mann-Whitney U and Kruskal-Wallis H tests were used for non-normally distributed quantitative variables. A P value ≤ .05 was considered statistically significant (10).

## Results

A comparison of clinical, laboratory, and radiological data between extrahepatic and intrahepatic NC groups is presented in Table 1. Patients with extrahepatic NC had significantly higher levels of alkaline phosphatase (ALKP) and γ-glutamyl transferase (GGT) compared with those with intrahepatic NC (P = .006 and P < .001, respectively).

**Table 1:** Comparison between extrahepatic and intrahepatic NC groups regarding clinical, laboratory and radiological data.

Clinical data	Extrahepatic NCNo (%) 46 (100)	Intrahepatic NCNo (%) 28 (100)	Test of significant.	P value
Sex Male	23 (50)	11 (39.3)	$x^{2}$ -0.508	0.370
Female	23 (50)	17 (60.7)		
Age (days)			τ=0.138	0.891
Min. – Max.	23 – 105	38 – 108		
Mean ± SD.	65.54 ± 19.02	64.93 ± 17.93		
Median (IQR)	62 (53 – 80)	61.50 (51 – 77)		
Abdominal US: Hepatomegaly	3(6.5)	0(0)	$x^{2}$ -1.390	FE_P_=0.285
Splenomegaly	1(2.2)	2(7.1)	$x^{2}$ -1.105	FE_P_ =0.553
GB contractility	3 (6.5)	2 (7.1)	$x^{2}-0.011$	FE_P_ =1.000
LFTs			U=633.50	0.907
T.Bil				
Min. – Max.	5 – 21	5.50 – 21		
Mean ± SD.	10.62 ± 3.33	10.90 ± 3.98		
Median (IQR)	10.5 (8.70 – 11.49)	10 (7.64 – 12.24)		
D.Bil			U=631.0	0.884
Min. – Max.	4.82 – 14.50	4.40 – 16		
Mean ± SD.	7.58 ± 2.04	7.98 ± 3.03		
Median (IQR)	7 (6 – 8.08)	7.02 (5.80 – 9.25)		
T.prot			t=1.180	0.242
Min. – Max.	4 – 6.50	3 – 6.40		
Mean ± SD.	5.46 ± 0.61	5.27 ± 0.79		
Median (IQR)	5.30 (5 – 6)	5.35 (6 – 5.90)		
Albumin			t=0.779	0.438
Min. – Max.	3 – 4.60	2.50 – 4.80		
Mean ± SD.	3.79 ± 0.39	3.71 ± 0.54		
Median (IQR)	4 (3.60 – 4)	3.75 (3.40 – 4)		
AST Min. – Max. Mean ± SD. Median (IQR)	100 – 500 247.8 ± 98.11 230.5 (174 – 329)	51 – 2222 398.6 ± 412 307 (182 – 486.5)	U=488.50	0.083
ALT Min. – Max. Mean ± SD. Median (IQR)	25 – 512 195.2 ± 108.3 184 (110 – 255)	23 – 2013 271.4 ± 384.6 177.5 (104 – 261)	U=640.50	0.969
ALKP Min. – Max. Mean ± SD. Median (IQR)			U=398.50	0.006^*^
	172 – 2176	171 – 736		
	614 ± 298.1	455 ± 174.9		
	556.5 (490 – 730)	449 (312 – 575.5)		
GGT			U=189.0^*^	<0.001^*^
Min. – Max.	126 – 1678	46 – 1436		
Mean ± SD.	820.1 ± 427.6	330.9 ± 369.5		
Median (IQR)	690.5 (497 – 1145)	222 (108 – 405)		
PT			t=1.547	0.126
Min. – Max.	10 – 14.30	10 – 14		
Mean ± SD.	11.34 ± 1.17	11.79 ± 1.27		
Median (IQR)	11 (10 – 12)	11.70 (11 – 12.65)		
INR			U=624.0	0.822
Min. – Max.	0.93 – 1.6	0.90 – 97		
Mean ± SD.	1.15 ± 0.11	4.56 ± 18.12		
Median (IQR)	1.16 (16 – 1.20)	1.15 (16 – 1.20)		

No: Number, %: Percent, LFTs: Liver function tests, T.Bil: Total bilirubin, D.Bil: Direct bilirubin, T.prot: Total protein, AST: Aspartate aminotransferase, ALT: Alanine aminotransferase, ALKP: Alkaline phosphatase, GGT: Gamma-glutamyl transferase, PT: Prothrombin time, INR: International normalized ratio, SD: Standard deviation,*: Significant U= Mann Whitney test , $x^{2}$: is the Chi square test, FE: Fisher Exact test.

Histopathological Assessment of NC Cases

**Fig. 1:**
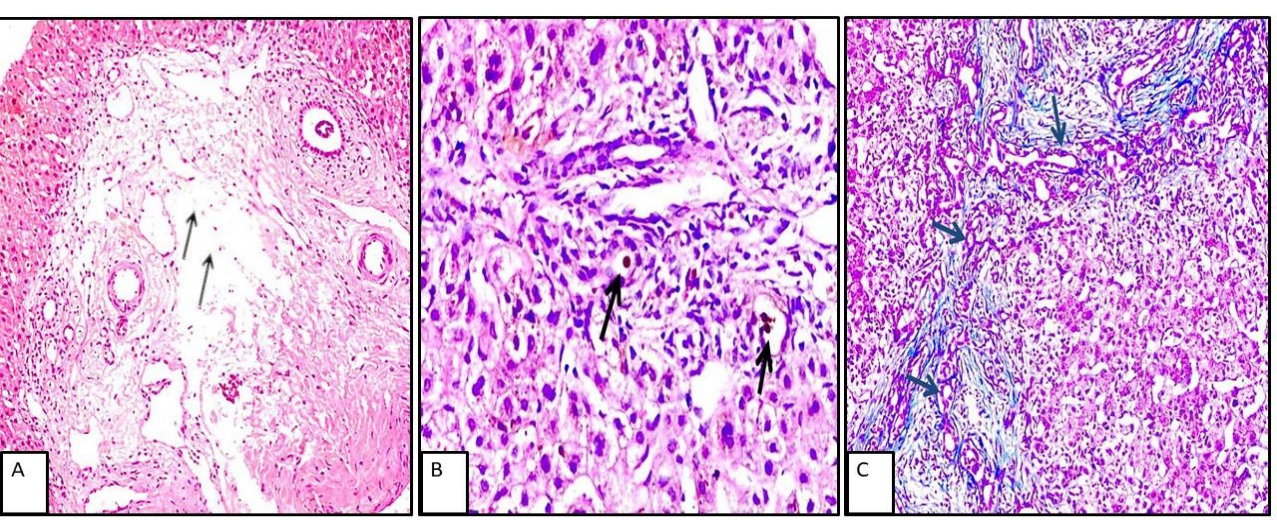
A case of extrahepatic NC, A: Portal tract edema black arrows (H&E x40), B: Intraluminal bile lugs black arrows, (H&E x200). C: Bile duct proliferation, black arrows (Masson trichrome x100).

**Fig. 2:**
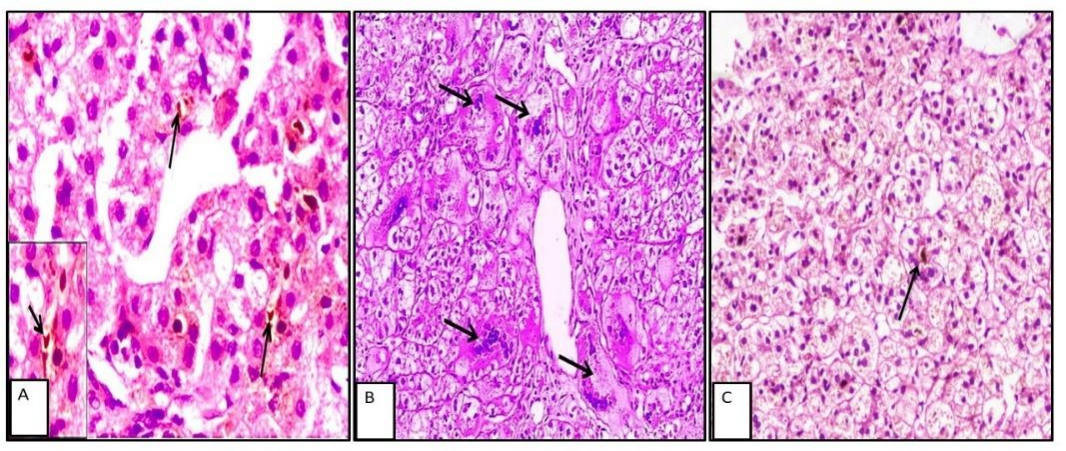
A: Case of intrahepatic NC with intracanalicular cholestasis black arrows, (H&Ex400). B: Case of neonatal hepatitis with prominent giant cell transformation, black arrows (H&E x400). C: Case of PFIC demonstrating cholestatic rosettes, red arrow (H&E x200).

In extrahepatic cholestasis, portal tract changes were more prominent, including periportal edema, bile plugs, and bile duct proliferation. In contrast, intrahepatic cholestasis was more frequently associated with parenchymal alterations such as cholestatic rosettes and intrahepatic or intracanalicular cholestasis (Fig. 1 and Fig. 2).

A comparison of histopathological parameters between extrahepatic and intrahepatic NC groups is presented in Table 2. Extrahepatic cholestasis was significantly associated with portal tract changes, including fibrosis, edema, bile plugs, bile duct proliferation, and bile ductular proliferation (P < .001 for all). Conversely, intrahepatic cholestasis was significantly associated with parenchymal features, including hepatocyte rosetting (P < .001), lymphocytic permeation (P = .001), extramedullary hematopoiesis (P = .002), steatosis (P < .001), and microabscess formation (P = .004).

### ATG5 Expression in Hepatocytes

**Fig. 3:**
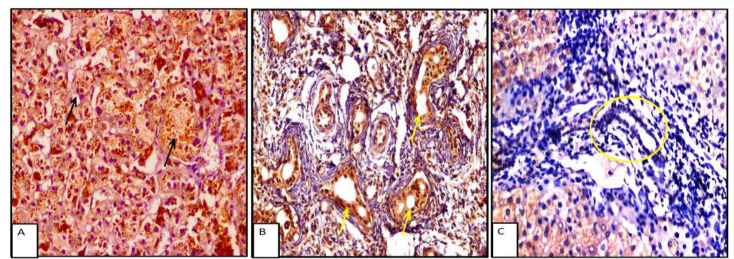
A: Strong zone II cytoplasmic staining of hepatocytes byATG5, black arrows (IHC x200). B: Strong staining of biliary epithelium byATG5, red arrows (IHC x200). C: Negative staining of biliary epithelium for ATG5, yellow circle (IHC x400).

All studied cases showed positive ATG5 expression in hepatocytes. In the extrahepatic NC group, the mean ± SD ATG5 H-score was 177.17 ± 51.50, whereas in the intrahepatic NC group, it was 204.29 ± 57.00 (Fig. 3). A significant difference in ATG5 expression was noted in zone II hepatocytes between extrahepatic and intrahepatic NC (P = .029). Moderate to strong ATG5 expression was observed in 31 patients (67.4%) with extrahepatic NC and in 23 patients (82.1%) with intrahepatic NC.

**Fig. 4:**
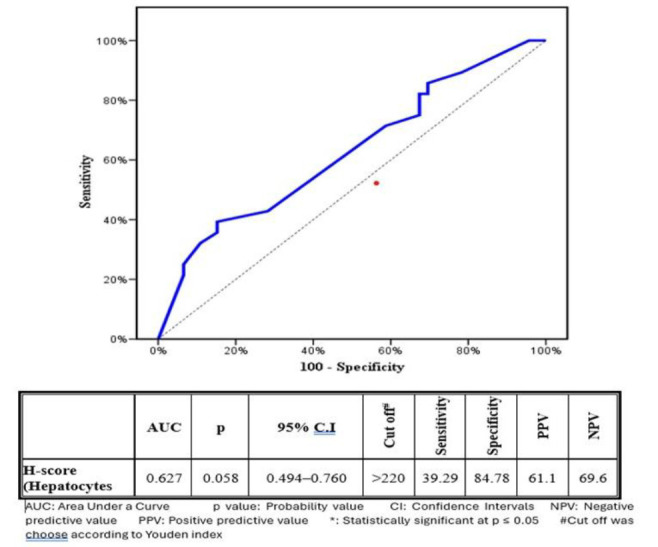
ROC curve for H-score (Hepatocytes)) to discriminate intrahepatic cases from extratrahepatic cases. Table: Validity (AUC, sensitivity, specificity) for H-score (Hepatocytes) to discriminate intrahepatic cases from extratrahepatic cases.

Receiver operating characteristic (ROC) curve analysis was performed to evaluate the diagnostic utility of ATG5 expression in differentiating between extrahepatic and intrahepatic cholestasis. Although the difference approached statistical significance (P = .058), higher ATG5 expression was consistently observed in hepatocytes from intrahepatic NC compared with extrahepatic NC. The specificity and sensitivity of ATG5 expression were 84.78% and 39.29%, respectively (Fig. 4).

### ATG5 Expression in Biliary Epithelium

No significant difference in ATG5 IRS expression was observed in biliary epithelium between extrahepatic and intrahepatic NC groups (Table 3).

**Table 2:** Comparison between extrahepatic and intrahepatic NC groups regarding the histopathological parameters

Parameters		Extra hepatic No (%) 46 (100)	Intra hepatic No (%) 28 (100)	$x^{2}$	p
Portal tract changes	Portal tract edema				
	Present	45 (97.8)	16 (57.1)	^19.893*^	FE_P_<0.001^*^
	Absent	1 (2.2)	12 (42.9)		
	Bile duct proliferation				
	Present	44 (95.7)	8 (28.6)	37.490*	<0.001^*^
	Absent	2 (4.3)	20 (71.4)		
	Bile ductular proliferation				
	Present	45(97.8)	10(35.7)	^35.187*^	<0.001^*^
	Absent	1 (2.2)	18 (64.3)		
	Bile plugs				
	Present	46 (100)	8 (28.6)	^45.026*^	<0.001^*^
	Absent	0(0)	20(71.4)		
	Degree of Lymphocytic infiltrate				
	Mild	41 (89.1)	27 (96.4)	1.244	FE_P_ =0.399
	Moderate	5 (10.9)	1 (3.6)		
	Degree of Neutrophil infiltrate Mild	17 (37)	11 (39.3)	1.071	0.301
	Moderate + Marked	15 (32.6)	5 (17.9)		
	Degree of Eosinophil infiltrate Mild	32 (69.6)	16 (57.1)	1.179	0.278
	Moderate + Marked	14 (30.4)	12 (42.9)		
	Hepatocyte swelling				
	Present	38 (82.6)	26 (92.9)	1.564	FE_P_ =0.301
Parenchymal changes	Absent	8 (17.4)	2 (7.1)		
	Rosetting				
	Present	6 (13)	13 (46.4)	14.066*	<0.001^*^
	Absent	40(87)	15(53.6%)		
	Giant cell transformation				
	Present	25 (54.3)	18 (64.3)	0.706	0.401
	Absent	21 (45.7)	10 (35.7)		
	Lymphocytic permeation				
	Present	2 (4.3)	10 (35.7)	12.604*	FE_P_ =0.001^*^
	Absent	44 (95.7)	18 (64.3)		
	Extra medullary hematopoiesis				
	Present	9 (19.6)	15 (53.6)	9.185*	0.002^*^
	Absent	37 (80.4)	13 (46.4)		
	Steatosis				
	Present	1 (2.2)	8 (28.6)	11.353*	FE_P_ =0.001^*^
	Absent	45 (97.8)	20 (71.4)		
	Microabscess				
	Present	4 (8.7)	10 (35.7)	8.283*	0.004^*^
	Absent	42 (91.3)	18 (64.3)		

$x^{2}$ : Chi square test

MC: Monte Carlo

FE: Fisher Exact

p: p value for comparison between extra hepatic and intra hepatic NC.

*:Statistically significant at p ≤ 0.05

**Table 3:** Comparison between extrahepatic and intrahepatic NC groups regarding ATG5 expression.

ATG5 expression		Hepatic		Test of Sig.	P value
		Extra hepatic No = (%) 46 (100)	Intra hepatic No = (%) 28 (100)		
Hepatocytes	Hepatocyte Expression Positive	46 (100)	28 (100)	NA	
	Negative	0(0)	0(0)		
	H-score				
	Min. – Max.	80 – 280	120 – 280	U= 480.0	0.063
	Mean ± SD.	177.17 ± 51.50	204.29 ± 57.05		
	Median	180	180		
	Zone I intensity				
	Mild	2 (4.3)	0 (0)	$x^{2}$ =2.427	MC_P_=0.326
	Moderate	30 (65.2)	15 (53.6)		
	Strong	14 (30.4)	13 (46.4)		
	Zone II intensity				
	Mild	15 (32.6)	5 (17.9)	$x^{2}$ =7.086*	0.029^*^
	Moderate	27 (58.7)	14 (50)		
	Strong	4 (8.7)	9 (32.1)		
	Zone III intensity				
	Mild	32 (69.6)	17 (60.7)	$x^{2}$ =0.610	0.435
	Moderate	14 (30.4)	11 (39.3)		
	Strong	0 (0)	0 (0)		
Bile ducts	Expression Positive Negative	40 (87) 6 (13)	27 (96.4) 1 (3.6)	$x^{2}$ =1.823	FE_P_ =0.242
	Intensity Mild Moderate	25 (62.5) 15 (37.5)			
			17 (63)	$x^{2}$ =0.001	0.969
			10 (37)		
	Percentage				
	Min. – Max.	10 – 80	10 – 80	U= 467.0	0.342
	Mean ± SD.	42.75 ± 23.53	374 ± 24.31		
	Median	40	40		
	IRS Positive (>1-6) Negative ( ≤1)	25 (54.3) 21 (45.7)	15 (53.6) 13 (46.4)	$x^{2}$ =0.004	0.948

IQR: Inter quartile range

SD: Standard deviation *: Significant U= Mann Whitney test,$x^{2}$: is the Chi square test FE: Fisher Exact test No: Number %: Percent

*H-score: Histoscore *IRS: Immune reactivity score NA: None applicable

### Relationship Between Hepatocyte ATG5 Expression and Clinicopathological Parameters

In the extrahepatic NC group, overexpression of ATG5 in hepatocytes was significantly associated with absent gallbladder contractility (P = .005) (Table 4). Furthermore, hepatocyte ATG5 overexpression was significantly correlated with mild portal tract fibrosis (P = .038) and mild portal tract lymphocytic infiltrates (P = .005) (Table 5).

### Correlation of Hepatocyte ATG5 H-Score With Laboratory Parameters

**Fig. 5:**
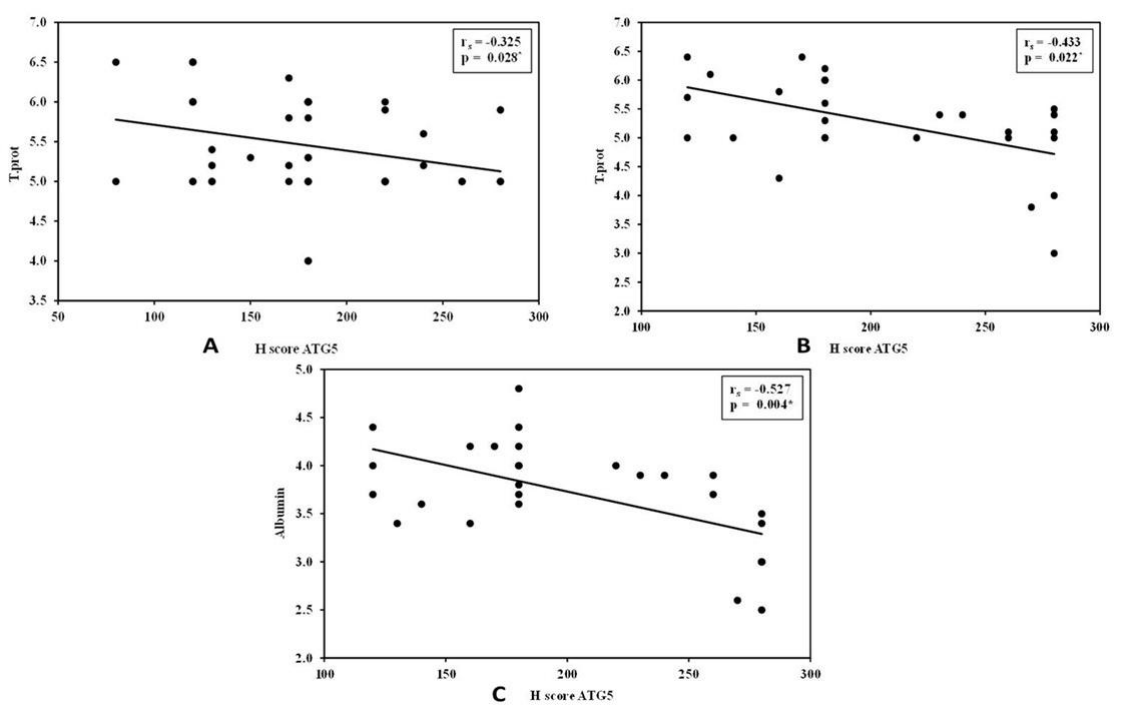
(A): Correlation between ATG5 H -score and total protein in extrahepatic NC group. (B): Correlation between ATG5 H score and total protein in intrahepatic NC group (C): Correlation between ATG5 H- score and albumin in intrahepatic NC group.

Correlation analysis between hepatocyte ATG5 H-scores and laboratory findings is illustrated in Figure 5. In the extrahepatic NC group, a significant negative correlation was observed between hepatocyte ATG5 H-score and total protein levels (r = –0.325, P = .028). In the intrahepatic NC group, significant negative correlations were detected between hepatocyte ATG5 H-score and both total protein (r = –0.433, P = .022) and albumin levels (r = –0.527, P = .004).

**Table 4:** Relationship between ATG5 H-score expression in hepatocyte and radiological data in both extrahepatic and intrahepatic cholestasis groups.

	H-score (Hepatocytes) ATG5 expression				
	**Extra hepatic**	**U testp value**		**Intra hepatic**	**U testp value**
	**Mean ± SD.**			**Mean ± SD.**	
Hepatomegaly					
Present	--	NA		160 ± 34.64	21.0(0.248)
Absent	177.17 ± 51.50			209.60 ± 57.34	
Splenomegaly					
Present	170	NA		180	22.0(0.762)
Absent	177.33 ± 52.07			206.15 ± 58.86	
GB contractility					
Contracted	106.67 ± 23.09	8.500(0.005^*^)		175.0 ± 7.071	17.500(0.476)
Absent	182.09 ± 49.36			206.54 ± 58.65	

*H-score: Histoscore *SD: Standard deviation *: Significant U= Mann Whitney test.

NA: None Applicable

**Table 5:** Relationship between ATG5 H-score expression in hepatocytes and histopathological parameter in both extrahepatic and intrahepatic NC groups.

Parameters		ATG5 H-score expression in EHC	Test of Significant	P value	ATG5 H-score expression in IHC	Test of Significant	P value
		Mean ± SD.			Mean ± SD.		
Portal tract changes	Degree of Fibrosis Mild fibrosis Moderate fibrosis	203.57 ± 59.69 165.63 ± 43.62	U=138.50	0.038^*^	200.50 ± 59.60 191.67 ± 39.20	H=4.027	0.134
	Portal tract edema Present Absent	177.11 ± 528 180^#^	NA	–	212.50 ± 58.14 193.33 ± 56.14	U= 82.0	0.537
	Bile duct proliferation Present Absent	174.77 ± 50.23 230 ± 70.71	U= 19.500	0.213	215 ± 62.79 200 ± 55.72	U= 68.500	0.566
	Bile ductular proliferation Present Absent	174.89 ± 49.66 280^#^	NA	-	208.0 ± 63.91 202.22 ± 54.72	NA	-
	Bile plugs Present Absent	177.17 ± 51.50 –	NA	-	232.50 ± 61.12 193 ± 52.73	U= 52.0	0.165
	Degree of Lymphocytic infiltrate Mild Moderate	184.63 ± 47.86 116 ± 40.99	U= 27.500^*^	0.005^*^	205.19 ± 57.94 180^#^	NA	–
	Degree of Neutrophil infiltrate Mild Moderate + Marked	189.41 ± 50.56 164.67 ± 52.90	U=94.0	0.216	205.45 ± 46.55 184 ± 79.25	H=1.181	0.554
	Degree of Eosinophil infiltrate Mild Moderate + Marked	181.88 ± 543 166.43 ± 45.17	U=198.0	0.527	207.50 ± 62.13 200 ± 51.87	U=90.0	0.802
Parenchymal changes	Hepatocyte swelling Present Absent	1765 ± 49.95 182.50 ± 61.82	U= 134.50	0.618	202.31 ± 578 230 ± 70.71	U= 17.0	0.476
	Resetting Present Absent	178.75 ± 49.57 166.67 ± 67.43	U= 93.500	0.397	226.92 ± 56.18 184.67 ± 51.81	U= 55.0	0.052
	Giant cell transformation Present Absent	168.0 ± 52.99 188.10 ± 48.64	U=190.50	0.106	196.67 ± 55.62 218 ± 59.96	U=71.50	0.382
	Lymphocytic permeation Present Absent	171.50 ± 55.37 154 ± 44.50	U=40.0	0.852	187 ± 401 213.89 ± 63.63	U=65.500	0.245
	Extra medullary hematopoiesis Present Absent	170 ± 70.71 177.50 ± 51.54	U= 96.500	0.051	190.67 ± 40.79 220 ± 69.88	U= 71.500	0.235
	Steatosis Present Absent	143.33 ± 42.13 185.41 ± 50.64	NA	–	203.75 ± 58.54 204.50 ± 57.99	U= 78.0	0.940
	Microabscess Present Absent	260^#^ 175.33 ± 50.52	U= 66.0	0.510	197 ± 51.43 208.33 ± 61	U= 80.500	0.654

SD: Standard deviation

U: Mann Whitney test

H: H for Kruskal Wallis test

p: p value for Relation between H-score (Hepatocytes) ATG5 expression and different parameters

*: Statistically significant at p ≤ 0.05 *H-score: Histoscore *

EHC: Extrahepatic cholestasis,

IHC; Intrahepatic cholestasis.

NA: None Applicable.

### Relationship Between Biliary Epithelium ATG5 IRS and Clinicopathological Parameters

The associations between biliary epithelium ATG5 immunoreactivity score (IRS) and radiological as well as histopathological parameters in both extrahepatic and intrahepatic NC groups are summarized in Tables 6 and 7. No statistically significant correlations were identified in either group.

In both study groups, no significant associations were detected between ATG5 expression in the biliary epithelium and radiological findings.

In the extrahepatic NC group, overexpression of ATG5 in the biliary epithelium was significantly associated with the absence of extramedullary hematopoiesis (P = .036). In the intrahepatic NC group, ATG5 overexpression was significantly associated with the presence of microabscesses (P = .031) (Tables 6 and 7).

### Correlation of Biliary Epithelium ATG5 IRS With Laboratory Parameters

**Fig. 6:**
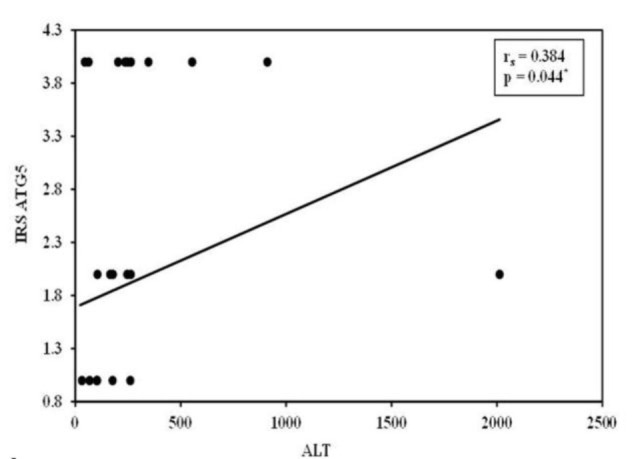
Correlation between ATG5 IRS and ALT level in intrahepatic NC group.

Correlation analysis is shown in Figure 6. In the extrahepatic NC group, no significant correlations were found between ATG5 expression in the biliary epithelium and laboratory parameters. In contrast, in the intrahepatic NC group, biliary epithelium ATG5 overexpression demonstrated a significant positive correlation with ALT levels (r = 0.384, P = .044).

### Correlation Between Hepatocyte ATG5 H-Score and Biliary Epithelium ATG5 IRS

In the extrahepatic NC group, a significant positive correlation was observed between hepatocyte ATG5 H-scores and biliary epithelium ATG5 IRS values (r = 0.595, P < .001). In contrast, no significant correlation was found between these parameters in the intrahepatic NC group (table not shown).

**Table 6:** Relationship between biliary epithelium ATG5 IRS and radiological parameters in both extrahepatic and intrahepatic cholestasis groups.

	ATG5 IRS expression			
	**Extra hepatic NC**	**U testP value**	**Intra hepatic NC**	**U testP value**
	**Mean ± SD.**		**Mean ± SD.**	
Hepatomegaly				
Present	–	NA	0.33 ± 0.58	12.50(0.062)
Absent	1.93 ± 1.54		2.12 ± 1.59	
Splenomegaly				
Absent	2	NA	0.50 ± 0.71	11.50(0.222)
Present	1.93 ± 1.56		2.04 ± 1.61	
GB contractility				
Contracted	0.33 ± 0.58	21.0(0.053)	2.0 ± 0.0	22.0(0.762)
Absent	2.05 ± 1.53		1.92 ± 1.67	

SD: Standard deviation

U: Mann Whitney test NA: none applicable

p: p value for comparing between different categories

*: Statistically significant at p ≤ 0.05

**Table 7:** Relationship between biliary epithelium ATG5 IRS and histopathological parameters in both extrahepatic and intrahepatic NC groups.

Parameters		IRS ATG5 expression of EHC	Test of Significant	P value	IRS ATG5 expression of IHC	Test of Significant	P value
		Mean ± SD.			Mean ± SD.		
Portal tract changes	Degree of Fibrosis Mild fibrosis Moderate fibrosis	2.07 ± 1.64 1.88 ± 1.52	U=210.0	0.730	2.05 ± 1.61 1.50 ± 1.52	H=0.481	0.786
	Portal tract edema Present Absent	1.89 ± 1.53 4.0	NA	–	2.00 ± 1.59 1.83 ± 1.70	U=88.50	0.732
	Bile duct proliferation Present Absent	1.95 ± 1.57 1.50 ± 0.71	U= 40.0	0.852	2.25 ± 1.58 1.80 ± 1.64	U= 66.0	0.500
	Bile ductular proliferation Present Absent	1.93 ± 1.56 2.0	NA	-	2.30 ± 1.57 1.72 ± 1.64	U=70.50	0.356
	Bile plugs Present Absent	1.93 ± 1.54 –	NA	-	1.75 ± 1.58 2.00 ± 1.65	U=74.0	0.784
	Degree of Lymphocytic infiltrate Mild Moderate	2.0 ± 1.53 1.40 ± 1.67	U= 78.50	0.408	1.85 ± 1.59 4.0	NA	–
	Degree of Neutrophil infiltrate Mild Moderate + Marked	1.94 ± 1.48 1.80 ± 1.42	U=126.50	0.970	1.73 ± 1.62 1.20 ± 1.64	U=21.50	0.510
	Degree of Eosinophil infiltrate Mild Moderate + Marked	1.94 ± 1.54 1.93 ± 1.59	U=222.0	0.961	1.81 ± 1.47 2.50 ± 1.72	H=4.637	0.098
Parenchymal changes	Hepatocyte swelling Present Absent	1.95 ± 1.58 1.88 ± 1.46	U= 150.50	0.966	1.77 ± 1.56 4.00 ± 0.00	U= 7.0	0.106
	Rosetting Present Absent	1.90 ± 1.55 2.17 ± 1.60	U= 106.50	0.667	2.31 ± 1.75 1.60 ± 1.45	U= 76.0	0.339
	Giant cell transformation Present Absent	1.68 ± 1.63 2.24 ± 1.41	U=199.50	0.151	1.94 ± 1.66 1.90 ± 1.60	U=89.50	0.981
	Lymphocytic permeation Present Absent	1.0 ± 1.41 1.98 ± 1.55	U=29.50	0.464	1.94 ± 1.66 1.90 ± 1.60	U=89.50	0.981
	Extra medullary hematopoiesis Present Absent	1.0 ± 1.32 2.16 ± 1.52	U= 91.50*	0.036^*^	1.93 ± 1.67 1.92 ± 1.61	U= 97.0	1.000
	Steatosis Present Absent	143.33 ± 42.13 185.41 ± 50.64	NA	–	2.00 ± 1.77 1.90 ± 1.59	U= 78.50	0.940
	Microabscess Present Absent	2.50 ± 1.73 1.88 ± 1.53	U= 67.0	0.534	2.80 ± 1.40 1.44 ± 1.54	U= 45.0	0.031^*^

SD: Standard deviation

U: Mann Whitney test

H: H for Kruskal Wallis test

p: p value for Relation between H-score (Hepatocytes) ATG5 expression and different parameters

*: Statistically significant at p ≤ 0.05 *H-score: Histoscore *

EHC: Extrahepatic cholestasis,

IHC; intrahepatic cholestasis.

## Discussion

Neonatal cholestasis (NC), characterized by persistent conjugated hyperbilirubinemia, remains a significant diagnostic challenge despite advancements in diagnostic modalities (11). The exact pathogenesis of biliary atresia (BA), the leading cause of NC, is still unclear (12).

In the present study, patients with extrahepatic NC had significantly higher ALKP and GGT levels. Similarly, Ağın et al reported that serum GGT levels were significantly higher in BA than in non-BA groups (P < .001) (13). Conversely, San et al found that markedly elevated GGT and ALKP levels may also occur in intrahepatic cholestasis caused by ductopenia, cholangitic congenital hepatic fibrosis, cytomegalovirus infection, PFIC type III, and inspissated bile syndrome (14). Both GGT and ALKP are recognized biochemical markers of cholestasis. Increased GGT activity is associated with primary and secondary hepatobiliary disorders, whereas sustained ALKP elevation is more often linked to liver and bone pathology (15). As bile acids accumulate in the liver, they disrupt hepatocyte membranes, releasing GGT and ALKP, while also stimulating ALKP synthesis (16). Elevated serum GGT is therefore considered a reliable marker of bile duct injury (17).

Histopathological assessment was particularly valuable in differentiating intrahepatic from extrahepatic cholestasis in this study. Extrahepatic cases were characterized by portal tract changes—fibrosis, edema, bile duct proliferation, ductular proliferation, and bile plugs—whereas intrahepatic cases were more frequently associated with parenchymal alterations, including extramedullary hematopoiesis (EMH). These findings are consistent with Bilal et al, who observed bile duct proliferation, bile plugs, periportal edema, and fibrosis in all BA patients (18). Portal fibrosis is thought to arise from hepatic stellate cell activation, which drives type I collagen deposition (19). Ductular proliferation may result from pro-inflammatory cytokine signaling, such as osteopontin, or from biliary differentiation of progenitor cells and trans-differentiation of periportal hepatocytes into biliary-type cells (20). Kandil et al also reported that EMH was absent or rare in most BA cases but significantly more frequent in non-BA patients (21). However, Russo et al found no difference between BA and non-BA cases regarding hepatocellular swelling and EMH (22). EMH may reflect residual fetal hepatic hematopoiesis or pathological responses to infection, tumors, anemia, or metabolic stress (23,24). Infection remains one of the most important causes of intrahepatic cholestasis (25).

Autophagy is a lysosome-mediated recycling pathway that maintains cellular homeostasis by degrading damaged organelles, proteins, and intracellular pathogens (4). Autophagy dysfunction has been implicated in diverse liver diseases, including NAFLD, alcoholic liver disease, drug-induced injury, cholestasis, viral hepatitis, and hepatocellular carcinoma (4). In cholestatic liver disease, the role of autophagy is complex and appears to depend on timing (early inflammation vs later bile acid injury) and cell type (hepatocytes vs bile duct epithelium) (26).

Our findings demonstrated significantly higher ATG5 expression in zone II hepatocytes in intrahepatic NC. This is notable, as hepatocyte repopulation in zones I and III is modulated by proliferation originating from zone II (27). Evidence suggests autophagy is essential for maintaining liver progenitor cell (LPC) function. Cheng et al demonstrated that LPCs exhibit higher autophagic activity than differentiated hepatocytes, and ATG5 knockdown impaired LPC self-renewal, proliferation, and hepatic differentiation (28). Furthermore, inhibition of autophagy sensitized LPCs to senescence, while Ma et al reported that ATG5 suppression severely impaired LPC differentiation (29).

We also observed significant associations between hepatocyte ATG5 overexpression and reduced serum total protein and albumin levels. Cholestasis results from impaired bile formation or excretion (30). Prolonged hepatocellular dysfunction reduces albumin synthesis, leading to hypoalbuminemia, a common finding in chronic liver disease (31). Experimental models show that autophagy stimulation during cholestasis reduces liver injury (26). In BA, autophagosome accumulation and elevated mitophagy markers in hepatocytes further support autophagy induction (32). ATG5 plays a central role in protein homeostasis. Its loss, or that of other autophagy regulators such as ATG7, results in endoplasmic reticulum stress, mitochondrial dysfunction, oxidative stress, and impaired protein synthesis (33). mTORC1, a major negative regulator of autophagy, promotes protein synthesis but is itself modulated by autophagy-related proteins, including p62/SQSTM1, which interacts with mTOR-raptor to regulate amino acid sensing (34,35). Thus, autophagy can indirectly self-regulate by modulating mTORC1 and protein synthesis (36).

In extrahepatic NC, hepatocyte ATG5 overexpression was significantly associated with absent gallbladder contractility. This aligns with previous studies showing that gallbladder abnormalities—absence, small size, irregular morphology, abnormal wall thickness, and lack of contraction—are reliable ultrasound features for diagnosing BA (37–39). Embryologically, the hepatic diverticulum gives rise to the liver, intrahepatic and extrahepatic bile ducts, cystic duct, and gallbladder (40). The Notch signaling pathway, essential for biliary morphogenesis, regulates cholangiocyte differentiation from LPCs. Notch deficiency leads to bile duct malformation and cholestasis requiring liver transplantation (41). Autophagy is critical for maintaining biliary epithelium homeostasis and progenitor cell function (42). Notably, autophagy inversely regulates biliary differentiation via mTOR-Notch signaling: mTOR inhibition (e.g., by rapamycin or nutrient deprivation) enhances autophagy, suppressing Notch signaling and biliary differentiation, whereas mTOR activation suppresses autophagy and promotes Notch-STAT3–mediated cholangiocyte differentiation (42).

The present study demonstrated a significant association between hepatocyte ATG5 overexpression in extrahepatic NC and the absence of portal tract fibrosis as well as the presence of only mild lymphocytic infiltrates. Autophagy may exert anti-fibrotic effects by inducing apoptosis of hepatic stellate cells (HSCs), facilitating degradation of pro-fibrotic mediators such as collagen and metalloproteinases, and reducing exosome-mediated profibrotic signaling (43).

ATG5 also plays a critical role in immune regulation. Innate lymphoid cells and natural killer (NK) cells require ATG5 for maturation and survival, particularly during homeostatic proliferation under lymphopenic conditions (44). Similarly, ATG5 deficiency has been shown to reduce thymic cellularity and peripheral T-cell counts by enhancing apoptosis, underscoring its importance in T-cell homeostasis (45).

In the present study, overexpression of ATG5 in the biliary epithelium showed a significant positive correlation with serum ALT levels. Elevated ALT is a marker of hepatocellular injury, ranging from minor to severe (46). In the liver, tumor necrosis factor α (TNFα) is a common mediator of hepatocellular death. Autophagy prevents TNFα-induced injury by inhibiting caspase-8 activation and the mitochondrial apoptotic pathway, suggesting that autophagy may serve as a therapeutic target in TNF-dependent tissue injury (47).

Within the intrahepatic NC group, biliary epithelium ATG5 overexpression was significantly associated with the presence of microabscesses. Autophagy has been shown to exert protective effects in sepsis by modulating macrophage polarization and suppressing inflammasome activation (48). Under septic conditions, autophagy activation via ATF4 protects liver function (49), which may explain this association.

In contrast, in the extrahepatic NC group, ATG5 overexpression in biliary epithelium was significantly associated with the absence of extramedullary hematopoiesis (EMH). Autophagy-related proteins are critical for hematopoiesis (50), and hematopoietic cell–specific ATG5 deficiency results in lymphopenia, anemia, and survival defects (51).

The present study also revealed significant positive correlations between hepatocyte ATG5 H-scores and biliary epithelium ATG5 IRS values in the extrahepatic NC group. This finding may reflect crosstalk between resident and non-resident liver cells during cholestatic injury. Multiple cell types, including cholangiocytes, HSCs, portal fibroblasts, and vascular cells, interact in the setting of cholestasis to drive liver damage and fibrosis (52). Cholangiocyte injury, in particular, initiates a cascade of cellular signaling that promotes ductular reaction, biliary and bridging fibrosis, and eventual progression to chronic liver disease and cirrhosis (52).

Cholestasis induces hepatic bile acid accumulation, which disrupts transport and homeostatic mechanisms, promoting fibrosis through interactions with portal fibroblasts and HSCs. Angiogenesis is closely linked to biliary fibrosis, with angiocrine signaling between portal fibroblasts, HSCs, and endothelial cells contributing to disease progression. Furthermore, immune cell infiltration during cholestasis and cholangiopathies exacerbates fibrogenesis, with different immune subsets exerting distinct effects on portal fibroblasts, HSCs, and cholangiocytes (53).

## Conclusion

In extrahepatic NC, portal tract changes were more prominent, whereas in intrahepatic NC, parenchymal alterations predominated. ATG5 expression may serve as a useful adjunctive marker, alongside histopathological features, to help distinguish intrahepatic from extrahepatic NC.

Our findings suggest that ATG5 may play a dual role in NC. In extrahepatic NC, ATG5 overexpression appeared protective, being associated with only mild fibrosis and mild inflammation. In contrast, in intrahepatic NC, ATG5 overexpression was linked to elevated ALT levels, suggesting a potential contribution to hepatocellular injury. The preferential overexpression of ATG5 in zone II hepatocytes further supports its role in maintaining hepatic regeneration and protecting against injury.

Taken together, ATG5 may represent a promising surrogate diagnostic marker and a potential therapeutic target in neonatal cholestasis.

## Data Availability

There is no additional data separate from available in cited references.
